# The readiness of public primary health care (PUSKESMAS) for cardiovascular services in Makasar city, Indonesia

**DOI:** 10.1186/s12913-022-08499-w

**Published:** 2022-09-01

**Authors:** Dian Sidik Arsyad, Esliana Fitrida Hamsyah, Nurul Qalby, Andriany Qanitha, Jan Westerink, Maarten J. Cramer, Frank L. J. Visseren, Pieter A. Doevendans, Ansariadi Ansariadi

**Affiliations:** 1 Department of Cardiology, Division of Heart and Lungs, University Medical Center Utrecht, University of Utrecht, 3584 CT Utrecht, The Netherlands; 2 Department of Epidemiology, Faculty of Public Health, Hasanuddin University, Makassar, Indonesia; 3 Faculty of Medicine, Hasanuddin University, Makassar, Indonesia; 4 Department of Vascular Medicine, University Medical Center Utrecht, Utrecht, The Netherlands; 5 Netherlands Heart Institute Utrecht, Utrecht, The Netherlands

**Keywords:** Primary health care, Service readiness, WHO-SARA, Cardiovascular disease, Puskesmas, Indonesia

## Abstract

**Backgrounds:**

The increasing burden of cardiovascular disease (CVD) has become a major challenge globally, including in Indonesia. Understanding the readiness of primary health care facilities is necessary to confront the challenge of providing access to quality CVD health care services. Our study aimed to provide information regarding readiness to deliver CVD health services in public primary health care namely Puskesmas.

**Methods:**

The study questionnaire was adapted from the World Health Organization (WHO) Service Availability and Readiness Assessment (SARA), modified based on the package of essentials for non-communicable disease (PEN) and the Indonesian Ministry of health regulation. Data were collected from all Puskesmas facilities (*N* = 47) located in Makassar city. We analysed relevant data following the WHO-SARA manual to assess the readiness of Puskesmas to deliver CVD services. Human resources, diagnostic capacity, supporting equipment, essential medication, infrastructure and guidelines, and ambulatory services domain were assessed based on the availability of each tracer item in a particular domain. The mean domain score was calculated based on the availability of tracer items within each domain. Furthermore, the means of all domains’ scores are expressed as an overall readiness index. Higher scores indicate greater readiness of Puskesmas to deliver CVD-related health care.

**Results:**

Puskesmas delivers health promotion, disease prevention, and prompt diagnosis for cardiovascular-related diseases, including hypertension, diabetes, coronary heart disease (CHD), and stroke. Meanwhile, basic treatments were observed in the majority of the Puskesmas. Long-term care for hypertension and diabetes patients and rehabilitation for CHD and stroke were only observed in a few Puskesmas. The readiness score of Puskesmas to deliver CVD health care ranged from 60 to 86 for. Furthermore, there were 11 Puskesmas (23.4%) with a score below 75, indicating a sub-optimal readiness for delivering CVD health services. A shortage of essential medicines and a low capacity for diagnostic testing were the most noticeable shortcomings leading to suboptimal readiness for high-quality CVD health services.

**Conclusion:**

Close cooperation with the government and other related stakeholders is required to tackle the identified shortcomings, especially the continuous monitoring of adequate supplies of medicines and diagnostic tools to achieve better CVD care for patients in Indonesia.

**Supplementary Information:**

The online version contains supplementary material available at 10.1186/s12913-022-08499-w.

## Background

Cardiovascular disease (CVD) continues to be the top cause of death worldwide, including in Indonesia, and the number of deaths in the population caused by CVD has increased significantly in the past decade [1, 2]. In 2019, approximately 38% of the total causes of death per 100,000 population in Indonesia was CVD, which is higher than in the rest of the Southeast Asian region [2].

According to a nationwide study in Indonesia, the prevalence of coronary heart disease and stroke diagnosed by physicians varied across regions, ranging from 0.7 to 2.2% for coronary heart diseases and from 4.1 to 14.7% for stroke [3]. The rapid increase in the prevalence of risk factors for cardiovascular disease may lead to a high future incidence of CVD. The potential growth of a large high-risk population in the fourth most populated country in the world will, consequently, burden the health care system.

Primary health care (PHC) has been identified as a crucial component in the health care system [4, 5]. Strengthening PHC capacity to provide health promotion, basic prevention, treatment, and rehabilitation services for CVD is a proven strategy that lowers the burden on the health care system [5, 6]. In Indonesia, PHC is performed in both the public and private sectors [7]. Public PHC is managed by the government and represented by Community Health Centers (CHC) named Puskesmas. With their auxiliary networks, Puskesmas deliver primary care services for individuals and communities within their designated areas [7]. In 2020, approximately 10,203 Puskesmas were distributed throughout the country, with an average of approximately 30,000 individuals in each catchment area and fewer individuals in rural areas. Furthermore, there are an estimated 68,320 community empowered health care networks named *Pos pembinaan terpadu* (Posbindu) for early detection and monitoring of non-communicable diseases [8], making Puskesmas the cornerstone for delivering health care services, including cardiovascular care in the population.

Accurate information on the readiness of health services is necessary to identify gaps to further improve health care quality. The World Health Organization (WHO), in collaboration with the United States Agency for International Development (USAID), developed a methodology and set of tools called the Service Availability and Readiness Assessment (SARA) that aim to provide information for stakeholders regarding the performance of the health care system over time [9]. An assessment of the Puskesmas using the WHO-SARA focusing on CVD health care services may be of value to identify gaps and opportunities to strengthen PHC in the Indonesian health system. Therefore, our study aims to provide actual information regarding the readiness of Puskesmas to deliver CVD health care services to the population.

## Materials and Methods

### Study design and settings

**Fig. 1:**
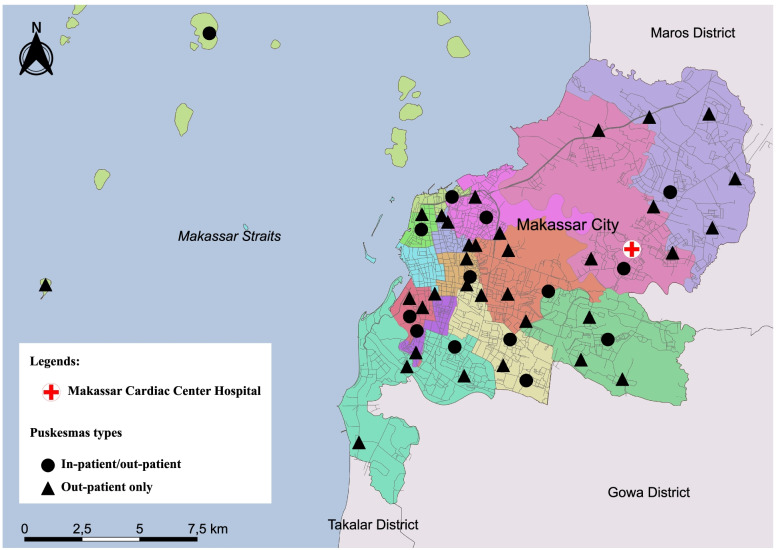
Geographical location and distribution of Puskesmas and Makassar Cardiac Center referral hospital

A facility-based cross-sectional study was conducted in Makassar city in South Sulawesi Province in Indonesia. Makassar is the largest city located in the eastern part of Indonesia, with approximately 175 km^2^, consisting of 15 sub-districts with 153 villages or *kelurahan*. According to the local statistics office, in 2020, 1.4 million people were currently residing in Makassar, making it the fifth most populated city in the country [10]. The geographic location of Makassar and the distribution of the Puskesmas are shown in Fig. 1.

### Population and Sample

We conducted a total sampling of all 47 public/government-owned primary health care facilities, namely, Puskesmas. In Indonesia, Puskesmas is the only primary health care facility managed by the government that is widely distributed and covers most of the population compared to private health care facilities. Furthermore, Puskesmas is the frontline facility for the population covered by national health insurance. In Indonesia, the Puskesmas are categorized into inpatient/outpatient and outpatient only facilities based on their ability to deliver overnight hospitalization care for observation and treatment. Currently, 47 Puskesmas provide primary health care and public health services for the population in Makassar. We approached All of the Puskesmas for interviews and observation. Our study encountered no refusals, resulting in a 100% response rate.

### Data Collection

To gather information related to the readiness of CVD care in the Puskesmas, we adapted and modified the WHO-SARA inventory by selecting only the relevant items for CVD health care and excluding all items for other diseases and programs (i.e., maternal and child health, infectious disease, and family planning). In addition, the modification is also based on the WHO Package of Essential Non-communicable (PEN) disease prevention checklists items for diagnostic tests and essential medicines for primary health care [11]. Furthermore, national regulation on Puskesmas and guidelines for managing CVD and its risk factors [12–14].

Following the modification, we validated the questionnaire content through a discussion with medical doctors and cardiologists to ensure that our instrument is sufficient to describe the readiness of Puskesmas. Training of the instrument and pilot testing was done to assure that our enumerator was familiar with the questionnaire content.

The data collection was divided into two parts. First, guided interviews were conducted with the appropriate informants, including the head of the facility, general practitioners, nurses, pharmacists, and laboratory technicians. In this stage, we gathered information related to facility statistics (e.g., number of overall patients with known CVD or risk factors including diabetes and hypertension, visits, the population coverage, distances to nearest and central referral facilities), human resources, CVD management-related training, and types of CVD health care service available in the Puskesmas. Second, we conducted observations using the modified WHO-SARA inventory checklist to capture facilities and infrastructures related to CVD health care in each Puskesmas.

Data were collected from March to June 2021 by research assistants who had been trained prior to the survey. The training aimed to ensure familiarity with the Puskesmas settings, study questions, indicators, and key informants for interviews. Puskesmas were visited 2–3 times during the data collection periods to anticipate the unavailability of the informants or documents required at the time of visits. We collected information regarding Puskesmas characteristics, including the number of beds, the number of *Posbindu* networks, and other relevant information from their annual reports. The modified WHO-SARA used in the current study is provided in the supplementary material (S1).

### Study variables

The WHO-SARA inventory is an instrument developed by the WHO to gather information from health care facilities regarding their capabilities to deliver general and specific health care [9]. In general, the tool sought to answer several questions, including about the availability of basic services offered by health care facilities, qualified health workers to deliver the services, and resources and support systems to guarantee high-quality services.

We categorized the available health care services in this study into (1) health services for hypertension and diabetes, including health promotion, screening and diagnosis, treatment, and monitoring (long-term care); and (2) health services for coronary heart disease (CHD) and stroke, including health promotion, screening and diagnosis, treatment, and rehabilitation.

We assessed the readiness of Puskesmas to deliver cardiovascular health care from the following domains: (1) human resources (specialist doctors, general practitioners, nurses, public health workers, pharmacists, and laboratory technicians); (2) diagnostic capacity (blood pressure, lipid profile, blood glucose, hemoglobin (Hb), creatinine, and blood urea nitrogen level); (3) supporting equipment (ECG, sphygmomanometer, anthropometric measurements, and CT scans); (4) essential CVD medicine (antiplatelet drugs, anticoagulants, statins, beta-blockers, angiotensin-converting enzyme (ACE)-inhibitor/angiotensin-receptor blockers (ARBs), and calcium channel blockers (CCB)); (5) infrastructure and guidelines (basic amenities including electricity, clean water, computers, internet connections, cellular lines, 24-hour hotlines, and CVD management guidelines); and (6) ambulatory services (ambulances and mobile home care).

### Data Analysis

Availability of the service was analysed by calculating the percentage of Puskesmas that delivers the listed services for CVD, namely health promotion screening and diagnosis, treatment, long-term care, and rehabilitation, to describe the availability of CVD health care in Makassar city.

Relevant data were analysed following the SARA manual of the WHO to assess the readiness of Puskesmas to deliver CVD service [9]. Items in each of the domains have equal weight. We calculated a cumulative number of items within each group divided by the total items and multiplied by 100 to acquire the domain score. The overall readiness score is calculated by adding each of the domains’ scores divided by the total number of domains. The readiness score ranges from 0 to 100, with higher scores interpreted as the facility having a better readiness for CVD health care services. The readiness score was compared to an agreed cutoff of 75, and the Puskesmas with a score above the cutoff were considered “optimal.”

Geographic distribution of the Puskesmas based on their readiness to deliver cardiovascular health care services in Makassar city is presented using maps. Data were analyzed using SPSS v.27 for the descriptive analysis and QGIS v.3.2 to visualize the data into thematic maps. All data were categorized by the types of Puskesmas, namely, inpatient/outpatient and outpatient only facilities.

### Research ethics

The current study was approved by the Institutional Review Board (IRB) at the Faculty of Public Health, Hasanuddin University, Indonesia, with the ethical approval number 2234/UN4.14.1/TP.02.02/2021. All informants gave their verbal informed consent prior to the interview and observation.

## Results

The general characteristics of the Puskesmas are shown in Table 1. In total, there were 47 Puskesmas in Makassar city, with one of the facilities recently constructed in 2019. There were only 35 outpatient (74%) and 12 inpatient/outpatient (26%) facilities. Every Puskesmas has its own defined working or coverage areas. On average, these facilities cover approximately 31,840 individuals living in 7171 households. The median distance to the nearest hospital was 2.5 km, and the median distance to the tertiary level referral facility, namely, Makassar Cardiac Center Hospital, was 9.7 km.

**Table 1:** General characteristics of public primary health care **(**Puskesmas**)** in Makassar

***General information***	Inpatient/ outpatient	Outpatient only	Overall
**Puskesmas characteristics**			
Number of Puskesmas	12	35	47
Average population/Puskesmas	33,490	31,795	31,840
Average household/Puskesmas	7873	6922	7171
Median distance to nearest hospital (km)	2.7	2.5	2.5
Median distance to MCC referral hospital	11.4	9.2	9.7
Average number of beds	18	–	n/a
Average community network (*Posbindu*)	10	8	9
**Puskesmas utilization**			
Average monthly patient visits			
Overall visits in 2020	2341	1523	1732
Percent changes from 2019	- 35%	- 41%	−39%
Hypertensive patients visits	120	95	101
Diabetic patients visits	59	44	48
CVD patients visits (CHD, stroke, etc.)	20	9	12

*MCC* Makassar Cardiac Center, *Posbindu* Integrated Supervisory Posts for Non-Communicable Disease, *CVD* Cardiovascular Diseases, *CHD* Coronary Heart Disease

According to the Puskesmas annual report, the estimates of monthly health care visits during 2020 varied across Puskesmas, with an average of 1732 visits/month. The number notably declined compared to 2019, with an average of a 39% reduction in visits/month. Furthermore, the average number of visits by patients with hypertension and diabetes mellitus was 101 and 48 visits/month, respectively. In contrast, the average number of patients with coronary heart disease, stroke, or other CVD visits in these facilities was 12 visits/month.

### Availability of cardiovascular health care services in Puskesmas

**Fig. 2:**
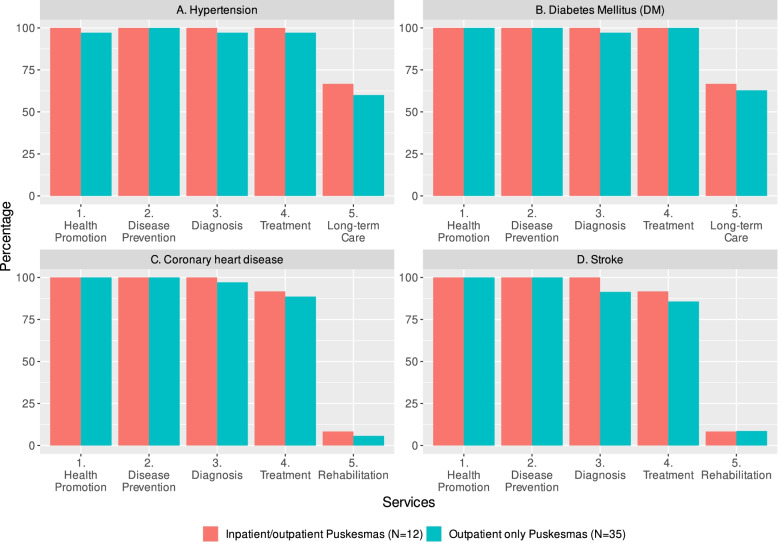
Available health care services for cardiovascular related diseases in Puskesmas

Figure 2 presents information related to health care services available for patients with cardiovascular-related diseases. Health promotion, disease prevention, and diagnosis for hypertension, diabetes mellitus, coronary heart disease and stroke were found in all (100%) inpatient/outpatient and most outpatient only Puskesmas (98.1%).

We found basic treatment services for hypertension and diabetes mellitus in more than 97% of Puskesmas, which was higher than services for coronary heart disease and stroke treatment found in less than 90% of Puskesmas. Meanwhile, less than 75% of Puskesmas provide long-term care for hypertension and diabetes, while very limited Puskesmas provide rehabilitation services for coronary heart disease and stroke.

### Readiness of cardiovascular health care service in Puskesmas.

Table 2 presents information regarding tracer items for assessing the readiness of Puskesmas to deliver cardiovascular health care. The average number of general practitioners and nurses working in Puskesmas was 2 and 8, respectively. Other health professionals, such as health educators, pharmacists, and laboratory technicians, were also available at all Puskesmas. Regarding diagnostic capacity, we found blood pressure, total cholesterol, blood glucose, and hemoglobin tests in all inpatient/outpatient and most outpatient Puskesmas. Meanwhile, high-density lipoprotein (HDL), low-density lipoprotein (LDL), hemoglobin A1C (HbA1c), and blood urine nitrogen tests for diagnosing CVD risk factors were not present in any Puskesmas.

**Table 2:** Puskesmas readiness related to cardiovascular health care

Readiness tracer items	Inpatient/ outpatient	Outpatient only	Overall
**Human resources; (**median (min-max))			
General practitioner	3 (1–5)	2 (0–4)	2 (0–5)
Nurses	12 (4–19)	6 (2–9)	8 (2–19)
Public health worker or health educator	2 (1–4)	2 (0–6)	2 (0–6)
Pharmacist	1 (1–2)	1 (1–2)	1 (1–2)
Laboratory technician	1 (1–2)	1 (0–1)	1 (0–2)
**Diagnostic test;** n (%)			
Blood Pressure	12 (100)	35 (100)	47 (100)
Blood Glucose	12 (100)	34 (97.1)	46 (97.9)
Hemoglobin (Hb)	12 (100)	33 (94.3)	45 (95.7)
Total Cholesterol	12 (100)	34 (97.1)	46 (97.9)
HDL Cholesterol	0 (0.0)	0 (0.0)	0 (0.0)
LDL Cholesterol	0 (0.0)	0 (0.0)	0 (0.0)
Hemoglobin A1c	0 (0.0)	0 (0.0)	0 (0.0)
Serum Creatinine	0 (0.0)	0 (0.0)	0 (0.0)
Blood Urea Nitrogen (BUN)	0 (0.0)	0 (0.0)	0 (0.0)
**Supporting equipment;** n (%)			
Sphygmomanometer	12 (100)	35 (100)	47 (100)
Weight scale	12 (100)	35 (100)	47 (100)
Height scale	12 (100)	35 (100)	47 (100)
Thermometer	12 (100)	35 (100)	47 (100)
Stethoscopes	12 (100)	35 (100)	47 (100)
Oxygen	12 (100)	35 (100)	47 (100)
ECG Machine	12 (100)	32 (91.4)	44 (95.6)
Glucometer POCT	10 (83.3)	32 (91.4)	42 (89.4)
Cholesterol POCT	12 (100)	34 (97.1)	46 (97.9)
Hemoglobin (Hb) meter POCT	11 (91.7)	27 (77.1)	38 (80.9)
CT Scan	0 (0.0)	0 (0.0)	0 (0.0)
**CVD essential medicine;** n (%)			
Statin (e.g., atorvastatin, simvastatin,)	5 (41.7)	22 (62.9)	27 (57.4)
Antiplatelet (e.g.,: Aspirin, clopidogrel)	6 (50.0)	11 (31.4)	17 (36.2)
Anticoagulation Drugs e.g., warfarin, heparin)	1 (8.3)	2 (5.7)	3 (6.4)
Beta-Blocker (e.g., propanolol)	6 (50.0)	10 (28.6)	16 (34.0)
ACE Inhibitor (e.g., kaptopril, lisinopril)	5 (41.7)	10 (28.6)	15 (13.9)
Calcium Channel blockers (e.g.,amlodipin)	12 (100)	35 (100)	47 (100)
**Infrastructure and guidelines;** n (%)			
Electricity	12 (100)	35 (100)	47 (100)
Water supply	12 (100)	35 (100)	47 (100)
Telephones (Hotline 24 hours)	11 (91.7)	34 (97.1)	45 (95.7)
Mobile Phones	12 (100)	35 (100)	47 (100)
Internet Connections	12 (100)	35 (100)	47 (100)
Guidelines for CVD management	9 (75.0)	28 (80.0)	37 (78.7)
**Ambulatory Services;** n (%)			
Patient home visits (Homecare)	11 (91.7)	34 (97.1)	45 (95.7)
Ambulance/Ambulatory Care	12 (100)	35 (100)	47 (100)

Values are presented as absolute numbers and percentages (%) or otherwise mentioned

*CVD* Cardiovascular disease, *HDL* High-density lipoprotein, *LDL* Low-density lipoprotein, *BUN* Blood Urea Nitrogen, *ECG* Electrocardiogram, *POCT* Point of care testing, *CT* Computed tomography, *ACE* Angiotensin converting enzyme

Standard supporting equipment related to CVD health services, namely, sphygmomanometers, weight and height scales, thermometers, stethoscopes, and oxygen devices, were available in all Puskesmas. Furthermore, electrocardiogram (ECG) machines, glucometers, hemoglobin point of care tests (POCT), and cholesterol POCTs were available in 95.6, 89.4, 97.9, and 80.9% of the Puskesmas, respectively. However, no CT scans were observed in the Puskesmas. In terms of CVD essential medicines, only calcium-channel blockers, namely, amlodipine for treating hypertension, were found in all Puskesmas. However, statins, antiplatelets, beta-blockers, ACE inhibitors, and anticoagulants were only found in 57.4, 36.2, 34.0, 13.9, and 6.4% of Puskesmas, respectively.

Basic amenities, namely, electricity and water supply, mobile phones and internet connections, were found in all Puskesmas. In addition, 37 Puskesmas (78.7%) had guidelines related to NCD management in general or CVD and risk factor management in particular available in the facility during the data collection.

**Fig. 3:**
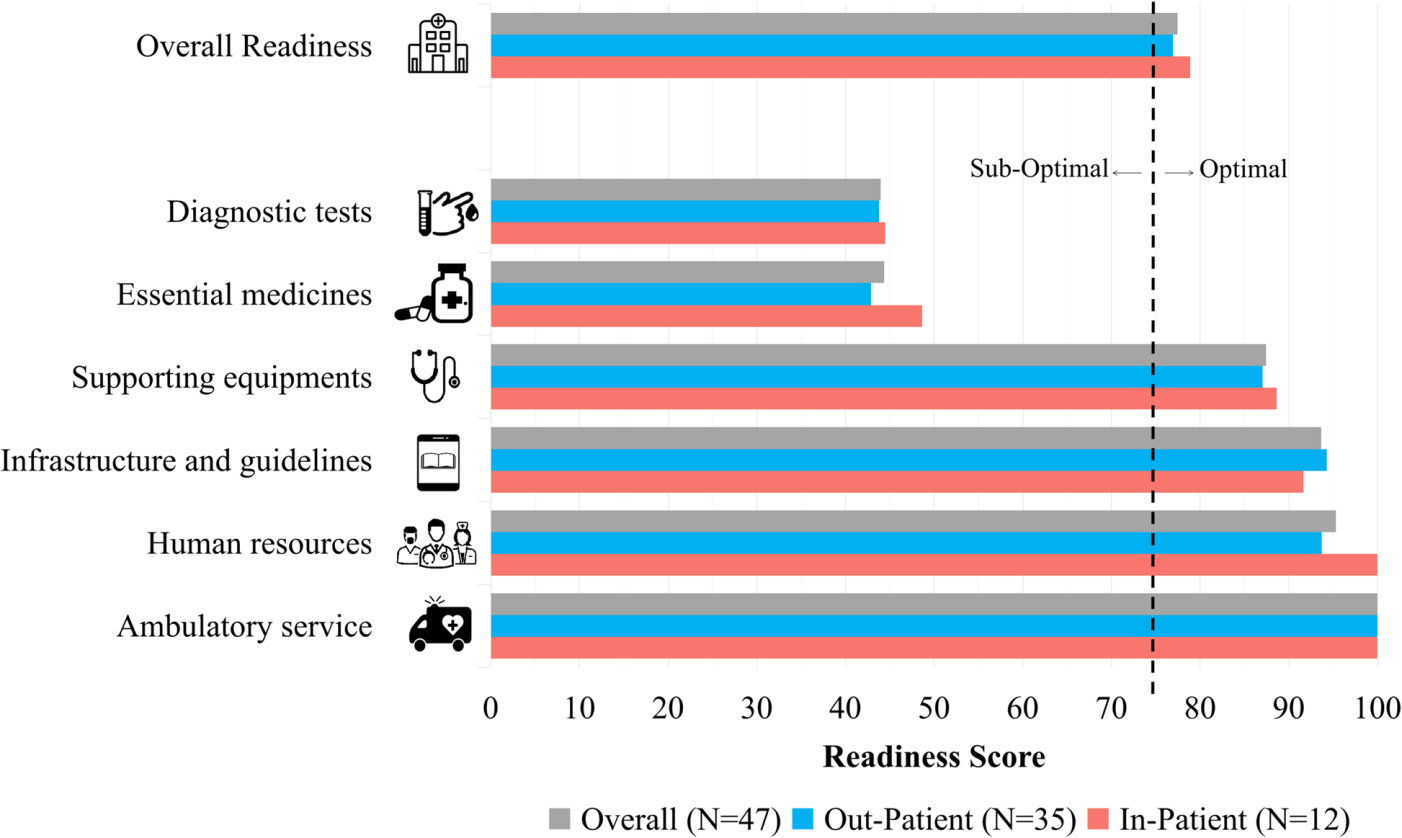
Readiness score of Puskesmas to deliver cardiovascular health care

**Fig. 4:**
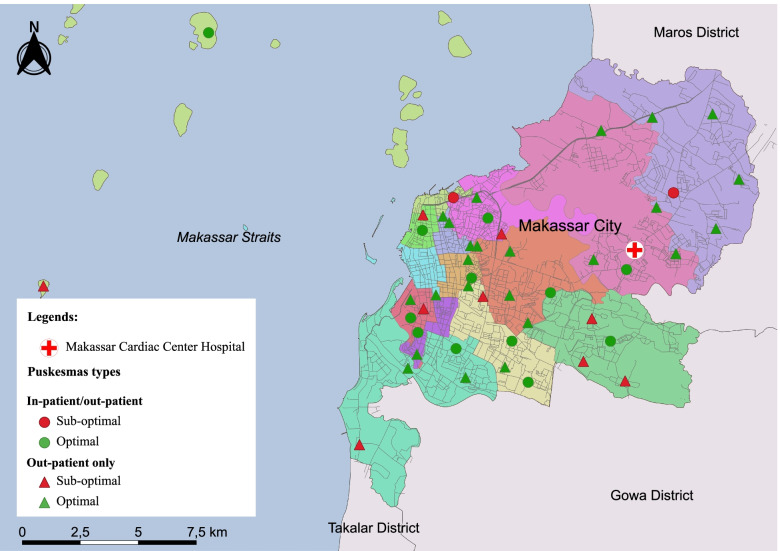
Distribution of Puskesmas based on their readiness score stratified by type

Figure 3 shows inpatient/outpatient and outpatient only Puskesmas average scores regarding their readiness in each domain. The highest score in the readiness domain for all Puskesmas was in ambulatory services, with a score of 100. Meanwhile, the lowest score from the readiness domain was demonstrated in the diagnostic capacity, followed by essential CVD medicines with scores of 43.9 and 44.3, respectively. Overall, the average readiness score for CVD health care services was 79 and 77 for inpatient/outpatient and outpatient only Puskesmas, respectively. The geographic distribution of Puskesmas according to their overall readiness score is shown in Fig. 4.

## Discussion

Our study aims to describe the readiness of Puskesmas to provide cardiovascular health care services to the population. In terms of health care service readiness, the average score from all of Puskesmas was 77.5. Although, in general, the score for Puskesmas in Makassar city indicated the readiness for CVD service was optimal, several Puskesmas were still categorized as sub-optimal. We observed that the availability of diagnostic tests and essential CVD medicines was the lowest among the six diagnostic domains used to assess facility readiness. Regarding the availability of primary care services, our study demonstrated that health promotion, primary prevention, screening, and basic diagnosis related to CVD were provided by the Puskesmas. On the other hand, not all Puskesmas provide treatment and long-term care for hypertension and diabetes or rehabilitation for coronary heart disease and stroke.

To date, ischemic heart disease, stroke, and diabetes are currently Indonesia’s leading causes of death [2]. The ability of health care systems to encounter these diseases and their risk factors is critical. As the front-line in delivering health care services, Puskesmas plays a significant role in preventing and controlling disease, including CVD, in the community. Although the prevalence of CVD and its risk factors in Indonesia are high in both primary and secondary prevention populations [15], our study showed a low number of patient visits per month for hypertension, diabetes and CVD, with decreasing trends for the average number of patient visits in 2020.

Despite decreased Puskesmas visits from the previous year due to the Covid 19 restrictions, over 1700 patient visits were reported in 2020, with only 10% being patients with CVD-related diseases (table 1). The lack of readiness from the primary health care facility to provide quality health services, including CVD-related health care, has been linked to patient utilization [16–18], possibly one of the reasons causing low utilization of Puskesmas for CVD services in our study population.

A review study in Southeast Asia also demonstrated the common barriers in primary health care service utilization for chronic illness, including CVD, involving a lack of diagnostic capacity and essential medicines in the facility [19]. Moreover, this also depicts the actual situation, including Indonesia’s PHC, where CVD patients tend to go to secondary care facilities for their CVD-related health care [16].

### Cardiovascular health care services in Puskesmas

According to the Indonesian Ministry of Health’s Regulation in 2019, the Puskesmas has the function of delivering community and individual health care, comprising health promotion, disease prevention, early diagnosis, and prompt treatment for health problems within their designated working areas [14]. Our findings demonstrated that all Puskesmas services are emphasized in primary prevention. Health promotion, screening, and early diagnosis of common NCD, including cardiovascular disease, are available despite their limitation in medication and laboratory testing capacity.

Primary prevention, which focuses on CVD risk factor prevention, is provided in all Puskesmas. An example of the disease prevention and risk factor control program in Puskesmas is the Chronic Disease Management Program (*Program Pengelolaan Penyakit Kronis* or PROLANIS) [20]. PROLANIS is a collaborative program between the Indonesian national health insurance system (INHI) implementing agency, the private sector and the primary health care system, including Puskesmas. The target population for the program is patients with hypertension and type 2 diabetes mellitus. Although the program positively impacted disease prevention and control, other studies identified some barriers during its implementation [20, 21].

In terms of treatment for risk factors, namely, hypertension and diabetes, Puskesmas deliver the essential medicines when available according to national regulations. However, they often lack most of the essential medications for CVD. The WHO issued a technical guideline for cardiovascular disease management to provide a set of practical, clinical interventions for strengthening CVD management and its risk factors in primary health [22].

Rehabilitation services for CVD patients in Puskesmas were very limited and mainly focused on the primary prevention of CVD, including those with developed risk factors, namely, hypertension, diabetes, and dyslipidemia. Meanwhile, secondary prevention patients, including post-myocardial infarction (MI) and stroke patients, had limited rehabilitation services available in the Puskesmas. With a growing number of CVD patients, secondary prevention and patient management after acute events are crucial. Despite the clear evidence, access to comprehensive rehabilitation services for CVD patients remains poor and underutilized, especially in primary health, which is a global problem, including in Indonesia [23]..

### Readiness for CVD health care services in Puskesmas

Health facility readiness is an important indicator in order to improve quality of the service delivery [9]. Although the WHO-SARA tool did not set a standard cut-off score for categorizing health care readiness, our study found that there were 23 (48.9%) Puskesmas in Makassar city having a readiness score below our agreed cut-off point of 75, indicating that the health service delivery related to CVD in these facilities was still suboptimal.

The WHO-SARA and service provision assessment (SPA) method and tool have been used in many countries to measure the readiness of health care facilities both for comprehensive care or specific to CVD care [24, 25]. A study in Nepal indicated a lower readiness score for CVD services in public health care facilities compared to the private sector [26]. Similar results from Bangladesh also showed a low average scores for WHO prioritized NCD related health services in primary health care sectors, which indicates a suboptimal readiness in delivering the disease related health care, including cardiovascular care [27]. Despite a different approach for defining readiness (i.e. lower cutoff points) with our study, many of these study demonstrated that health systems at the primary healthcare level especially in public sector are insufficiently prepared for CVD health services.

In terms of human resources, this study shows that general practitioners and nurses for managing clinical care, public health workers for health promotion and education, pharmacists and laboratory technicians for medicine supply and diagnostic tests were available, with the number of personnel varying among the Puskesmas. We observed that no medical specialist, namely, cardiologists or neurologists, was found in any Puskesmas. Our findings agreed with the Ministry of Health’s regulations concerning the minimum standard for human health resources in the Puskesmas, which only require that a general practitioner be available in the facilities [14]. In particular, for post-CHD and stroke patients, supervision for their secondary prevention can be managed by general practitioners and trained nurses when those patients are in a stable condition and do not exhibit any new symptoms. However, continuous training and a clear monitoring pathway are needed to ensure that patients with complications are referred promptly to secondary health care services.

Our findings also demonstrate that the diagnostic test capacity in the Puskesmas was limited. The ability to diagnose major risk factors for CVD with standard equipment was only applicable to hypertension with a sphygmomanometer and stethoscope, diabetes, and high total cholesterol with a point of care test (POCT). Meanwhile, low-density lipoprotein (LDL), high-density lipoprotein (HDL), creatinine, and blood urea nitrogen tests were not found in any Puskesmas, despite being regulated as the standard laboratory services in Puskesmas [28]. Findings from the national study involving public and private primary health care providers in Indonesia also indicated that only 67–81% of the facilities have the capacity to perform diagnostic services for common NCDs, including cardiovascular disease [29].

Recently, improving CVD diagnostic capacity using Tele-ECG for Puskesmas in Makassar was piloted to overcome limited human resources, screen for life-threatening events, improve early hospitalisation time, and prevent unnecessary hospital referrals [30].

The WHO has issued a list of essential medicines required in the health care systems for priority diseases, including CVD, to be available in functioning health systems at all times [31]. During the survey, we observed that many essential CVD medicines were unavailable at most Puskesmas. An inadequate supply of essential medicines for treating CVD and its risk factors is a common problem in the public sector compared to the private sector, especially in low-middle income countries (LMIC) settings, including Indonesia [32]. This situation is an ongoing challenge for health care systems that restrict the quality of care, especially for the population of a low socioeconomic level [33].

### Implications for primary health care system policy

Despite the optimal readiness of Puskesmas for delivering CVD health services, particularly in Makassar city in general, there are a few areas of concern that will need focus and corrective action to achieve a significant reduction in morbidity and mortality caused by CVD. The Ministry of Health has been implementing various programs to control the disease [34]. However, considering the substantial geographical inequalities between urban and rural areas, a comprehensive strategy to tackle this gap and provide sustainable, high-quality care is still a major challenge.

The findings of this study can help the government, policy-makers, and other stakeholders design appropriate interventions and programs to strengthen Puskesmas as the frontline in delivering CVD health care, particularly in the public sector.

### Strength and limitations

Our study was the first to explore the availability and readiness of Puskesmas as the main frontline in delivering quality CVD-related health services in the Indonesian primary health care setting. Several important limitations were identified in this study. Firstly, due to time and resource constraints, our study only includes public primary health care, in this case, the Puskesmas. Thus, the representativeness of our study was limited to public primary health care providers only, while at the same time, several private facilities also provide CVD care to our population. Secondly, the current study only describes the situation in Makassar city, an urban setting, while most of the people in Indonesia live in rural areas where the situation may differ from our findings. Thirdly, our study does not separate the general and CVD-specific readiness scores commonly used in other health care readiness studies. Lastly, the nature of the cross-sectional design of this study merely provides a screenshot of the conditions, not long-term evidence of the availability and readiness of the Puskesmas to deliver quality CVD health care services.

## Conclusions

Our study revealed that the overall readiness of the Puskesmas to deliver quality CVD health care services in Makassar was suboptimal. The limited capacity of diagnostic tests and the availability of essential CVD medicines were the most important shortcomings. Our analysis underscores that to improve the readiness of CVD health care services, the government needs to “fill in the gaps” by strengthening the poor aspects of the Puskesmas by continuous monitoring of adequate supplies in medicines and diagnostic testing infrastructure, as well as periodic training for health workers in managing CVD and its risk factors at the individual and community levels. Furthermore, extending CVD health services with appropriate rehabilitation services in Indonesian public primary health care is important.

## Supplementary Information

**Additional file 1.**

## Data Availability

Data will be available upon reasonable request from the corresponding author.

## Abbreviations

CVD: Cardiovascular diseases
SARA: Service availability and readiness assessment
Puskesmas: *Pusat kesehatan masyarakat* (public primary health care)
CHD: Coronary heart diseases
PHC: Primary health care
CHC: Community health center
Posbindu: *Pos pembinaan terpadu* (Integrated supervisory post for non-communicable disease)
ECG: Electrocardiogram
ACE: Angiotensin-converting enzyme
ARB: Angiotensin-receptor blockers
CCB: Calcium channel blockers
