# Supplementary material for: The readiness of public primary health care (PUSKESMAS) for cardiovascular services in Makasar city, Indonesia
Source: BMC Health Serv Res. 2022 Sep 1;22:1112. doi: 10.1186/s12913-022-08499-w (PMC9436735; doi:10.1186/s12913-022-08499-w)
Supplement: Supplementary file 1 — Additional file 1. [file 12913_2022_8499_MOESM1_ESM.pdf]

QUESTIONNAIRE / OBSERVATION CHECK LIST

READINESS FOR CARDIOVASCULAR DISEASE IN PUBLIC PRIMARY HEALTH CARE (PUSKESMAS)

Interview/Observation date : ___/______/_________ (hh/mm/yyyy)
Observer/Interviewer :
Duration : ___ Menit
Informant name :
Informant contact (HP) :

Puskesmas Information :

1. Puskesmas name :
2. Address :
3. Subdistrict/Village : /
4. Type of Puskesmas : In-patient and Outpatient / Out-patient only
5. Distance to nearest hospital : (Km)
6. Distance to MCC : (Km)
7. Number of beds : Beds (for in-patient puskesmas)
8. Geographic Location : ____ Latitude / ____ Longitude (DMS)
9. Number of POSBINDU :

No	Question	Responses			
**A. Population Coverage**					
A1	Number of Household in the working area	_______Household			
A2	Number of people living in the area	_____________ Persons			
A3	Number of registered participant covered by INHI	_____________ Persons			
**B. Resources**		**Number**		**Trained** (Training related to CVD and its risk factors)	
B1	Specialist physician (Total)				
B2	Cardiologist				
B3	Neurologist				
B4	General physcian				
B5	Dentist				
B6	Nurses				
B7	Public health / Health educator				
B8	Pharmacist				
B9	Laboratory technician				
**C. Number of patient visits per day (LB 1 PKM)**		**Previous Year**		**Last month**	**Average/month**
		**2019**	**2020**		
C1	Number of patient (total)				
C2	Hypertensive patients				
C3	Diabetes patients				
C4	CVD patients				
C5	Heart failure patients				
C6	Stroke patients				

D. Available tests		Methods of test /Type of device	Available (Y/N)
D1	Total Cholesterol		
D2	HDL Cholesterol		
D3	LDL Cholesterol		
D4	Blood Glucose		
D5	Haemoglobin (Hb) test		
D6	HbA1c		
D7	Creatinin		
D8	Ureum		
D9	Blood Pressure		

E. Available device		Available (Y/N)	Working (Y/N)	Number
E1	ECG Machine			
E2	Tensimeter (Digital/Manual)			
E3	Weight scale			
E4	Height scale			
E5	Thermometer			
E6	Stetoskop			
E7	Oxygen tube			
E8	Glucometer			
E9	CT Scan			
E10	Hb Meter			

F. Essential medicine		Available (Y/N)	Name of drugs
F1	Statin (e.g : Simvastatin, Atorvastatin, Pravastatin)		
F2	Antiplatelet Drugs (e.g. : Aspirin, Clopidogrel, Dipyridamole, Ticlopidine)		
F3	Anticoagulation Drugs (e.g.: Warfarin, Heparin, Enoxaparin, Edoxaban)		
F4	Beta-blocker (e.g. Bisoprolol, Propranolol)		
F5	ACE inhibitor / ARB (e.g: Lisinopril, kaptopril, Ramipril, dll)		
F6	Calcium Channeling Blockers (e.g : Amlodipin)		
F7	Others		

<table>
<tr><td colspan="7">Infrastructure and supporting equipment</td></tr>
<tr><td>G1</td><td colspan="2">Telephone (Hotline 24 hours)</td><td colspan="4">Yes / No</td></tr>
<tr><td>G2</td><td colspan="2">Mobile Phones / HP</td><td colspan="4">Yes / No</td></tr>
<tr><td>G3</td><td colspan="2">Internet</td><td colspan="4">Yes / No</td></tr>
<tr><td>G4</td><td colspan="2">Guideline books related to CVD treatment, management and controls</td><td colspan="4">Yes / No
(guideline name: ________________________________
________________________________</td></tr>
<tr><td colspan="3">G. Ambulatory service</td><td>Jumlah</td><td colspan="3">Working (Y/N)</td></tr>
<tr><td>H1</td><td colspan="2">Ambulance</td><td></td><td colspan="3"></td></tr>
<tr><td>H2</td><td colspan="2">Mobile Home Care</td><td></td><td colspan="3"></td></tr>
<tr><td colspan="7">I. Services Available</td></tr>
<tr><td colspan="2">Apakah puskesmas ini memberikan pelayanan terkait PJPD berikut</td><td>CVD Health promotion</td><td>CVD Prevention</td><td>CVD Diagnosis</td><td>CVD Treatment / Management</td><td>Long term care / Rehabilitation for CVD</td></tr>
<tr><td>I1</td><td>Non Communicable disease</td><td>(Yes/No)
Mention :</td><td>(Yes/No)
Mention :</td><td>(Yes/No)
Mention :</td><td>(Yes/No)
Mention :</td><td>(Yes/No)
Mention :</td></tr>
<tr><td>I2</td><td>Diabetes</td><td>(Yes/No)
Mention :</td><td>(Yes/No)
Mention :</td><td>(Yes/No)
Mention :</td><td>(Yes/No)
Mention :</td><td>(Yes/No)
Mention :</td></tr>
<tr><td>I3</td><td>Hypertension</td><td>(Yes/No)
Mention :</td><td>(Yes/No)
Mention :</td><td>(Yes/No)
Mention :</td><td>(Yes/No)
Mention :</td><td>(Yes/No)
Mention :</td></tr>
<tr><td>I3</td><td>Coronary heart disease</td><td>(Yes/No)
Mention :</td><td>(Yes/No)
Mention :</td><td>(Yes/No)
Mention :</td><td>(Yes/No)
Mention :</td><td>(Yes/No)
Mention :</td></tr>
<tr><td>I4</td><td>Stroke</td><td>(Yes/No)
Mention :</td><td>(Yes/No)
Mention :</td><td>(Yes/No)
Mention :</td><td>(Yes/No)
Mention :</td><td>(Yes/No)
Mention :</td></tr>
<tr><td>I6</td><td colspan="4">Puskesmas deliver referral service for emergency case for CVD (e.g.; Stroke or Heart attack/ Infark Myocard)</td><td colspan="2">Yes / No</td></tr>
<tr><td>I7</td><td colspan="4">On the last referal case, where do the patient referred to ?</td><td colspan="2">Health facility name
(______________________)</td></tr>
</table>
